# Development of Visual Detection of African Swine Fever Virus Using CRISPR/AapCas12b Lateral Flow Strip Based on Viral Major Capsid Protein Gene *B646L*

**DOI:** 10.3390/ani15223274

**Published:** 2025-11-12

**Authors:** Wanglong Zheng, Weilin Hao, Yajing Chang, Wangli Zheng, Can Lin, Zijian Xu, Xilong Kang, Nanhua Chen, Jianfa Bai, Jianzhong Zhu

**Affiliations:** 1 College of Veterinary Medicine, Yangzhou University, Yangzhou 225009, China; wanglongzheng@yzu.edu.cn (W.Z.); mz120231722@stu.yzu.edu.cn (W.H.); mx120241045@stu.yzu.edu.cn (Y.C.); dx120240222@stu.yzu.edu.cn (W.Z.); lc2068118712@outlook.com (C.L.); zijianxu111@outlook.com (Z.X.); 006954@yzu.edu.cn (X.K.); hnchen@yzu.edu.cn (N.C.); 2 Joint International Research Laboratory of Agriculture and Agri-Product Safety, Yangzhou 225009, China; 3 Comparative Medicine Research Institute, Yangzhou University, Yangzhou 225009, China; 4 Jiangsu Co-Innovation Center for Prevention and Control of Important Animal Infectious Diseases and Zoonoses, Yangzhou University, Yangzhou 225009, China; 5 Kansas State Veterinary Diagnostic Laboratory, Kansas State University, Manhattan, KS 66506, USA; jbai@vet.k-state.edu

**Keywords:** African swine fever virus (ASFV), *B646L*, AapCas12b, lateral flow strip (LFS), recombinase polymerase amplification (RPA)

## Abstract

African swine fever (ASF), induced by the African swine fever virus (ASFV), is an acute hemorrhagic disease characterized by high fever, systemic hemorrhages, and elevated mortality. Current diagnostic techniques including PCR and ELISA present limitations in field applications due to requirements for specialized equipment and prolonged processing duration. Therefore, rapid and accurate detection of ASFV has become a key link in ASF prevention and control. This study established a rapid and precise visual diagnostic approach by integrating the CRISPR/AapCas12b system with lateral flow strip (LFS) technology, specifically targeting the *B646L* gene encoding the major capsid protein p72. The CRISPR/AapCas12b-LFS platform achieved a sensitivity threshold of 6 copies/µL for *B646L* gene detection, completing analysis within an hour. Validation study confirmed exceptional specificity against common porcine pathogens including PRRSV, CSFV, PRV, PPV4, and PCV3. The developed assay demonstrated complete concordance with real-time PCR results when analyzing 34 clinical specimens including three heart samples, three liver samples, three spleen samples, three lung samples, three kidney samples, three lymph node samples, five serum samples, five blood samples, and five oral swab samples for ASFV detection. Overall, this method is sensitive, specific, and practicable onsite for ASFV detection, showing a great application potential for monitoring ASFV in the field.

## Simple Summary

Numerous diagnostic techniques for African swine fever virus identification have been established, including virus isolation, PCR, and serological assays including enzyme-linked immunosorbent assay. However, these methods require expensive instruments and professional operators, making them less suitable for on-site applications. In this study, we established an RPA-CRISPR/AapCas12b-LFS method for the detection of African swine fever virus by selecting the *B646L* gene as the target. This method achieved a sensitivity threshold of 6 copies/µL for *B646L* gene detection, completing analysis within an hour. It can be used for visual detection of African swine fever virus in clinical samples and has potential significance for early monitoring, prevention, and control of African swine fever.

## 1. Introduction

African swine fever (ASF), an acute and highly contagious hemorrhagic disease, affects both domestic and wild suids, with its etiological agent being African swine fever virus (ASFV) [1]. ASFV, a large and structurally complex double-stranded DNA virus, exhibits a genome size ranging from 170 to 190 kilobases (kb), encoding approximately 160 putative proteins [2,3]. As the sole representative of the Asfivirus genus within the Asfarviridae family, ASFV induces acute clinical manifestations including elevated body temperature, lethargy, loss of appetite, cutaneous discoloration, spleen enlargement, and hemorrhagic manifestations, typically resulting in catastrophic mortality rates [4,5]. Numerous diagnostic techniques for ASFV identification have been established, including virus isolation, PCR, and serological assays including enzyme-linked immunosorbent assay (ELISA) [6,7]. The existing gold standard for ASFV detection recommended by the World Organization for Animal Health (WOAH) is virus isolation, but the isolation and culture of ASFV must be carried out in an animal biosafety level 3 laboratory (ABSL-3) or above, which is time consuming and labor intensive [8]. These methods require expensive instruments and professional operators, making them less suitable for on-site applications. Therefore, developing new, convenient, simple, accurate, and inexpensive methods for detecting ASFV is the current and future development direction.

In recent years, clustered regularly interspaced short palindromic repeat (CRISPR)-associated Cas proteins have shown significant potential for rapid and sensitive nucleic acid detection [9]. Cas12b uniquely combines cis-cleavage (target DNA-specific cutting) and trans-cleavage (non-specific ssDNA degradation) activities [10]. Cis-cleavage of Cas12b refers to the sequence-specific cleavage of double-stranded DNA (dsDNA) guided by the sgRNA [11]. Trans-cleavage of Cas12b can efficiently cleave non-specific single stranded DNA (ssDNA), including DNA modified with fluorescent and quenching groups (FQ), producing detectable fluorescence signals [12]. By utilizing this characteristic, the CRISPR/Cas system has been combined with isothermal amplification technology to develop a series of simple, rapid, sensitive, and visual nucleic acid detection methods that do not require special precision instruments.

The *B646L* gene, encoding the viral major capsid protein p72, is highly conserved and well characterized. This target is widely used in both nucleic acid detection and phylogenetic analysis [13,14]. In this study, we established an RPA-CRISPR/AapCas12b-LFS method for the detection of ASFV by selecting the *B646L* gene as the target and optimizing a specific combination of RPA primers, sgRNA, and probes. Further, our research results indicated that the *B646L*-RPA-CRISPR/AapCas12b-LFS method can be effectively applied to clinical ASFV detection, providing a new detection method for clinical ASF prevention and control.

## 2. Materials and Methods

### 2.1. Reagents and Specimens

The Hipure Blood DNA Mini Kit (D3111-02) and virus DNA extraction kit Hipure Tissue DNA Mini Kit (D3121-02) were purchased from Magen Biotechnology Co., Ltd. (Guangzhou, China). The BL21 (DE3) competent *E.coli* (CB105) was purchased from Tiangen BioTech Co., Ltd. (Beijing China). The DEPC-treated Water (DNase, RNase free, R0021) and His-tag Protein Purification Kit (p2226) were bought from Beyotime Biotech, Inc. (Shanghai, China). The T7 High Yield RNA Transcription Kit (TR101), FastPure Gel DNA Extraction Mini Kit (DC301), and ClonExpress Ultra One Step Cloning Kit V2(C116) were both from Nanjing Vazyme Biotech Co., Ltd. (Nanjing, China). Spin Column RNA Cleanup & Concentration Kit (B518688) was purchased from Sangon Biotech Co., Ltd. (Shanghai, China). The recombinant polymerase amplification (RPA) kit (TwistAmp Basic, Cat #TABAS03KIT) was purchased from TwistDx Limited (Maidenhead, UK). The lateral flow strip (31203) was purchased from TOBOLIO Biotech Co., Ltd. (Shanghai, China). The CRISPR/AapCas12b reaction buffer (HOLMES Buffer for Cas12b, 32,043) was purchased from Tolo Biotech Co., Ltd. (Shanghai, China). The ASFV *B646L* gene standard plasmid was prepared and stored in our laboratory. The method for constructing the plasmid pCE-*B646L* is as follows. Porcine pseudorabies virus (PRV) attenuated strain Bartha-K61 and classical swine fever virus (CSFV) attenuated strain CVCC AV1412 were obtained from Jiangsu Nannong Hi Tech Co., Ltd. (Nanjing, China). Inactivated porcine parvovirus (PPV4, S-1 strain) was sourced from Shandong Huahong Biological Engineering Co., Ltd. (Binzhou, China). Porcine circovirus type 3 (PCV3) samples and porcine reproductive and respiratory syndrome virus (PRRSV) were isolated and cryopreserved in our laboratory.

### 2.2. Cloning of AapCas12b Gene, Protein Expression, and Purification

The AapCas12b gene sequence was PCR-amplified from plasmid pAG001 His6-TwinStrep-SUMO-AapCas12b (Addgene) (Watertown, MA, USA) using specific primers pET28a-AapCas12b-F/R (Table 1). We used the homologous recombination method to clone the Aap Cas12b gene between the ECOR I and Xho I sites of the PET28a vector. The pET28a-AapCas12b plasmid was transformed into BL21 (DE3) competent cells and subjected to expansion culture. Expression of the AapCas12b protein was induced with 0.5 mM IPTG at 16 °C and 220 rpm for 18 h. Induced bacterial cultures were ice-incubated for 30 min, and cells were harvested by centrifugation at 8000× *g* for 15 min at 4 °C. After discarding the supernatant, the wet cell pellet was weighed and resuspended thoroughly in pre-chilled non-denaturing lysis buffer (50 mM NaH_2_PO_4_, 300 mM NaCl, pH 8.0) at a ratio of 5 mL buffer per gram of wet cell weight. Bacterial cells were disrupted by sonication, and the lysate was centrifuged at 12,000× *g* for 30 min at 4 °C; the supernatant was retained. Ni-NTA resin was added to the supernatant and incubated at 4 °C for 2 h with gentle agitation. The resin-protein mixture was washed three times with 1 mL of wash buffer (50 mM NaH_2_PO_4_, 300 mM NaCl, 2 mM imidazole, pH 8.0) per wash. Target protein was eluted six times with 500 μL aliquots of elution buffer (50 mM NaH_2_PO_4_, 300 mM NaCl, 50 mM imidazole, pH 8.0) per elution step. The eluted Aap Cas12b protein was dialyzed three times against dialysis buffer (50 mM Tris-HCl, 600 mM NaCl, 2 mM DTT, 5% glycerol, pH 7.5) for 2 h each time. Finally, the protein was collected, aliquoted, and stored at −80 °C. In both the SDS-PAGE Coomassie brilliant blue staining and anti-His antibody, Western blotting confirmed the successful purification of AapCas12b protein.

### 2.3. Preparation of sgRNA

Based on the conserved *B646L* gene sequence obtained through multiple alignment, as well as the fixed scaffold sequence and the special protospacer adjacent motif (PAM) sequence 5′-TTN-3′ (where N can be any base of A, T, G, C) of AapCas12b, the encoding sequences for sgRNA targeting *B646L* gene were designed (Table 1). A total of 14 ASFV genomes, including six genotype I, seven genotype II, and one I/II recombinant strain, were aligned using Geneious primer software (version 2025.2). sgRNA primers were designed using the Benchling web-based software (https://www.benchling.com). The alignment results showed that there was no mismatch under the sgRNA. The DNA template of sgRNA was attached with the T7 promoter sequence (GAAATTAATACGATACTATATATAGGG) (Table 1) and then synthesized by Qingke Biotechnology Co., Ltd. (Tianjin, China) as the primers. The complementary primers were annealed in annealing buffer into double stranded (ds) DNA, followed by DNA extraction and purification. The purified dsDNA was used as a template to be transcribed into sgRNA through in vitro transcription (IVT). The transcribed sgRNA was purified using a column RNA rapid concentration and purification kit (Sangon Biotechnology Co., Ltd.) (Shanghai China).

### 2.4. B646L-Mediated CRISPR/AapCas12b Reaction

The *B646L* DNA sequence was based on the ASFV YZ-1 genome (GenBank No: ON456300), and the plasmid pCE-*B646L* constructed in our laboratory was used as the template DNA for reaction. The CRISPR/AapCas12b reaction system was established as follows: 1 μL AapCas12b (250 ng/μL), 1 μL ssDNA probe (4 μM, a 12-base-pair random DNA sequence with FAM as a reporter and BHQ1 as a quencher, as shown in Table 1), 1 μL sgRNA (150 ng/μL), 2 μL buffer, 1 µL plasmid pCE-*B646L* (100 ng/μL) or 2 μL *B646L* PCR amplified fragment (110 ng/μL), and add distilled water to a total volume of 20 µL. The reaction mix was incubated at 60 °C for 15 min for AapCas12b-mediated cleavage to occur. Amplification products were validated via sequential 1% agarose gel electrophoresis, followed by visualization under blue light, UV illumination, and fluorescence-based detection.

### 2.5. Recombinase Polymerase Amplification (RPA)

Following the design criteria for RPA primers, a length range of 28–35 base pairs and optimal amplicon sizes between 150 and 200 bp, two pairs of PRA primers targeting the *B646L* gene were designed (Table 1). The reaction protocol followed the TwistAmp Basic Kit (TwistDx) specifications, with each 50 μL amplification mixture comprising a 29.5 μL rehydration buffer, reaction beads, 0.48 μM forward/reverse primers, purified DNA template, 2.5 μL MgAc solution, and sterile water to adjust volume. Thermal incubation at 39 °C in a water bath for 15–20 min facilitated DNA amplification, with subsequent electrophoretic analysis of *B646L* gene products. Primer pairs underwent comparative evaluation to select optimal performers, with the chosen *B646L*-RPA amplicons subsequently integrated into CRISPR/AapCas12b detection workflows.

### 2.6. Establishment of RPA-CRISPR/AapCas12b-Lateral Flow Strip (LFS) Detection Method

Firstly, the biotin-conjugated, single-stranded DNA probe (5′-6-FAM-TTTTTTTATTTTTTT-biotin-3′) was commercially synthesized. Subsequently, colloidal gold particles functionalized with FAM-specific antibodies were conjugated to the ssDNA probe, forming a lateral flow detection complex with 5′-gold-antibody-FAM-ssDNA-biotin-3′ architecture. The amounts of reaction components are as follows: a 2 μL aliquot representing 10% of the RPA product, 1 μL AapCas12b enzyme, 1 μL sgRNA complex, 2 μL reaction buffer, 1 μL of 20 nM gold-biotin ssDNA probe, and 12.5 µL buffer, totaling 20 µL. Following a 15 min incubation at 60 °C, the LFS absorbent pad was immersed in the reaction mixture. The concentration of AapCas12b is 200 ng/uL. After complete saturation of the nitrocellulose membrane within 1–2 min, the control (C) line’s gold signal confirmed test validity. Visual interpretation was performed by assessing colloidal gold accumulation at the test (T) line position, with positive results indicating ASFV target presence in analyzed samples. In the presence of ASFV target sequences, the CRISPR/AapCas12b complex cleaves the ssDNA reporter probe, dissociating FAM-labeled gold nanoparticles from biotin molecules. Streptavidin immobilized on the control line (C) retains the biotin components, while liberated FAM-gold complexes migrate laterally to be captured by the second antibody at T line.

### 2.7. Sensitivity and Specificity of B646L-RPA-AapCas12b-LFS Detection Method

To assess the sensitivity of the detection, the standard plasmid pCE-*B646L* was diluted to different copy numbers, and continuous dilutions of plasmids with concentrations from 6 × 10^10^ copies/µL to 6 × 10^0^ copies/µL were prepared. RPA was performed on each plasmid dilution, followed by CRISPR/AapCas12b-LFS detection, and each dilution was tested in triplicate. The minimum copy number of the target pCE-*B646L* plasmid was detected with test strip to determine the sensitivity.

For specificity, viral nucleic acids including DNA or RNA were extracted from the other swine virus samples. RNA, extracted from porcine reproductive and respiratory syndrome virus (PRRSV) and classical swine fever virus (CSFV), was converted into cDNA before detecting. DNA was extracted from porcine parvovirus (PPV4), porcine pseudorabies virus (PRV), and porcine circovirus types 3 (PCV3). The above cDNA or DNA were used as the target DNA to conduct the CRISPR/AapCas12b reaction mediated by *B646L* sgRNA followed by LFS detection to determine the reaction specificity of CRISPR/AapCas12b-LFS detection.

### 2.8. Detection of Clinical Samples by RPA-AapCas12b-LFS

In this study, the nucleic acid DNAs were extracted from various pig tissues including three hearts, three livers, three spleens, three lungs, three kidneys, three lymph nodes, five serum, five blood samples and six oral swabs by using High Tissue DNA Mini Kit (D3121-02) and High Blood DNA Mini Kit (D3111-02) according to the instructions. Target DNA substrates were subjected to *B646L* sgRNA-mediated CRISPR/AapCas12b cleavage, followed by LFS readout.

## 3. Results

### 3.1. The Expression and Purification of AapCas12b

**Figure 1:**
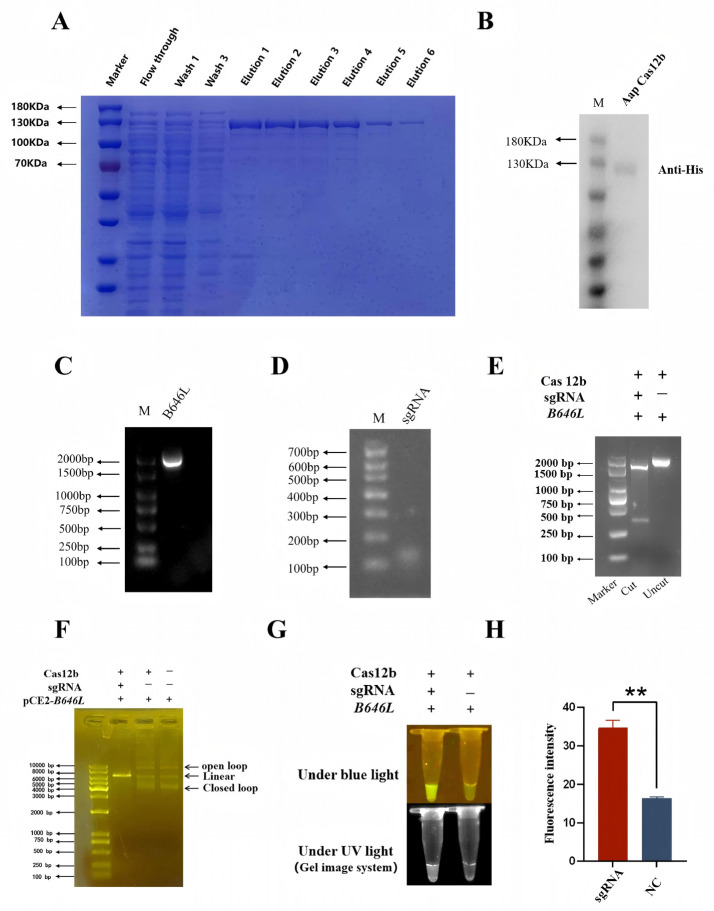
Expression and purification of AapCas12b protein and verification of its cleavage activities. (**A**) SDS-PAGE analysis Ni^2+^-NTA affinity chromatography purified His-labeled AapCas12b protein. (**B**) Western blotting detection of purified AapCas12b protein. (**C**,**D**) Analysis of *B646L* gene fragment of PCR products (**C**) and sgRNA (**D**) by agarose gel electrophoresis. (**E**,**F**) Verification of cis-cutting activity of AapCas12b protein on *B646L* gene fragment (**E**) and standard plasmid (**F**) by agarose gel electrophoresis. (**G**,**H**) Verification of trans-cleavage activity of AapCas12b protein, with the reaction detected under blue light and UV light (**G**), as well as by fluorescence signaling (**H**). NC, negative control without sgRNA. ** *p* < 0.01 by Student *t* test.

The AapCas12b gene was PCR amplified, and the PCR product was cloned into the pET28a vector by homologous recombination, followed by transformation into BL21 (DE3) competent *E. coli*. Purified AapCas12b was validated by SDS-PAGE/Coomassie blue staining for protein integrity, and anti-His antibody-based immunoblotting for target specificity. The results showed that the purified AapCas12b protein had a molecular weight of approximately 130 kDa (Figure 1A) and was recognized by a mouse anti-His tag monoclonal antibody (Figure 1B).

### 3.2. Validation of the Cleavage Activities of the Purified AapCas12b Protein

Cas12b uniquely combines cis-cleavage (target DNA-specific cutting) and trans-cleavage (non-specific ssDNA degradation) activities [10]. To verify the cis-cleavage and trans-cleavage activities of the purified AapCas12b protein, a DNA template of ASFV *B646L* PCR products (Figure 1C), a sgRNA of ASFV *B646L* (Figure 1D) were generated, and a ssDNA probe (Table 1) was synthesized. The purified AapCas12b protein could cleave the DNA template of ASFV *B646L* PCR products into two DNA segments (Figure 1E). Further, the purified AapCas12b protein could cleave the plasmid pCE-*B646L* into a linearized configuration (Figure 1F). The purified AapCas12b protein could also exhibit trans-cleavage activity to cleave ssDNA probe and generate a light signal (Figure 1G,H). These results suggested that the cis and trans-cleavage activities of purified AapCas12b protein are high, and the purified AapCas12b protein is feasible for subsequent detections.

### 3.3. Optimization of Reaction Conditions of CRISPR/AapCas12b Assay

**Figure 2:**
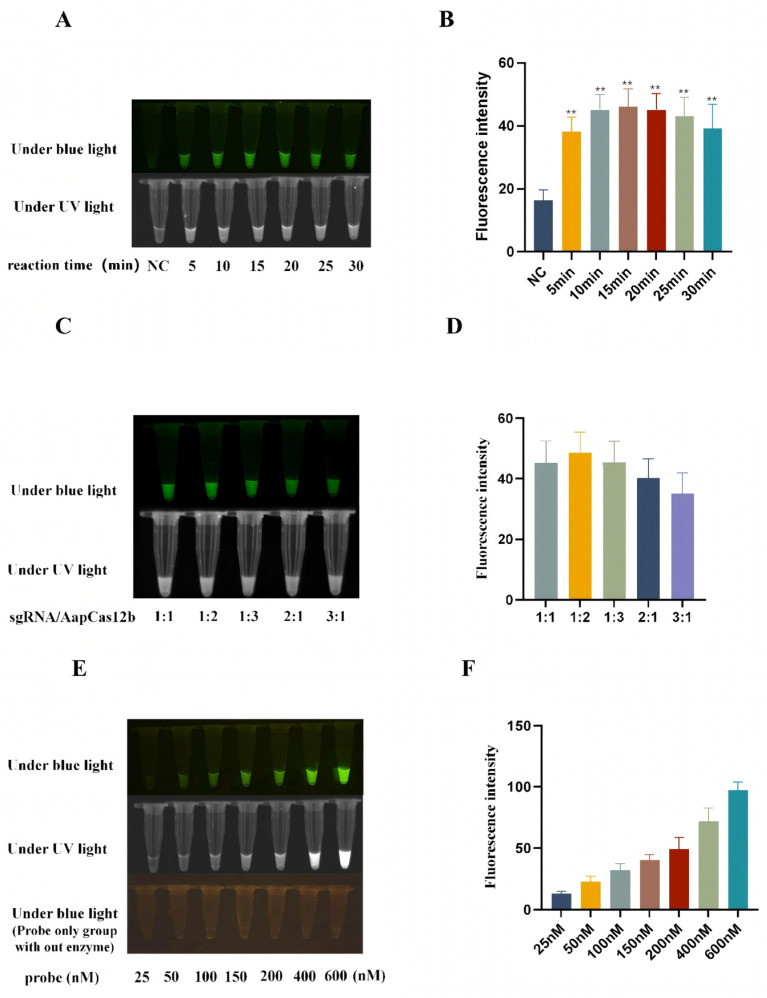
Optimization of reaction conditions of CRISPR/AapCas12b assay. (**A**) The reactions with different times as indicated were detected under blue light and ultraviolet light. (**B**) Green fluorescence signals were analyzed by ImageJ software(Version 1.52p). (**C**) The reactions with different sgRNA/Cas12b ratios were detected under blue light and ultraviolet light. (**D**) Green fluorescence signals were analyzed by ImageJ software. (**E**) The reactions with different probe concentrations were detected under blue light and ultraviolet light. (**F**) Green fluorescence signals were analyzed by ImageJ software. ** *p* < 0.05 versus negative control (NC) at 0 min by Student’s *t* test.

In order to optimize the reaction conditions including time, sgRNA/AapCas12b ratio, and probe concentration, the blue light, ultraviolet light, and fluorescence signals caused by the AapCas12b trans cutting of the ssDNA probe were analyzed. First, the CRISPR/AapCas12b reactions were performed for durations of 5, 10, 15, 20, 25, and 30 min, respectively. The results showed that when the reaction time was 15 min, the light signal was better than other time groups (Figure 2A,B). Second, the CRISPR/AapCas12b reactions were carried out at different ratios of sgRNA/AapCas12b (1:1, 1:2, 1:3, 2:1 and 3:1). The results have shown that the cutting activity of AapCas12b is affected by the sgRNA/AapCas12b ratio, and the signal of probe is higher at a 1:2 gRNA/AapCas12b ratio than at other ratios (Figure 2C,D). Third, the CRISPR/AapCas12b reactions were carried out with the increase in probe concentration from 25 nM to 600 nM. The results showed that the signal intensity of the blue light, ultraviolet light, and fluorescence signals all increased continuously (Figure 2E,F). A probe concentration of 150 nM was selected for its superior signal-to-noise ratio, ensuring robust detection performance.

### 3.4. Establishment of CRISPR/AapCas12b Mediated Lateral Flow Strip (LFS) Method

**Figure 3:**
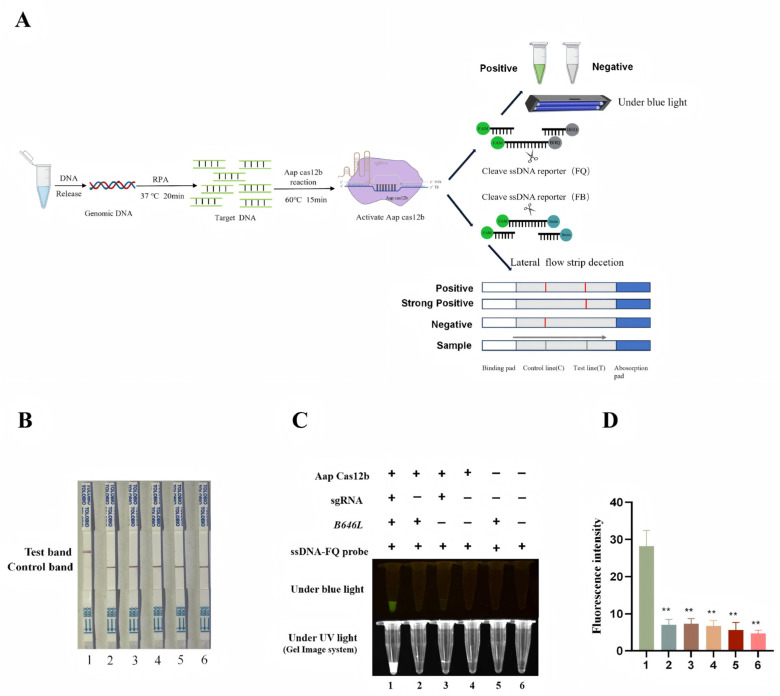
Establishment of CRISPR/AapCas12b mediated lateral flow test strip (LFS) method. (**A**) Schematic diagram of RPA-CRISPR/AapCas12b-LFS detection of ASFV. (**B**) The AapCas12b reactions were subjected to LFS detection. (**C**) The AapCas12b reactions were verified by blue light and UV light detection. (**D**) Green fluorescence signals were analyzed by ImageJ software. ** *p* < 0.01 versus No. 1 sample.

The green fluorescence of a probe can be observed under blue light, but a device that emits blue light is still needed, which is not convenient for field detection. In order to develop a convenient and efficient visual detection method, the CRISPR/AapCas12b mediated lateral flow strip (LFS) method was established. As shown in Figure 3A, the ssDNA probe labeled by 5′-FAM and 3′-biotin was used for LFS detection. The FAM antibody cross-linked gold particles on the test strip react with the FAM from probe. When the ASFV *B646L* gene is present in the test sample, the CRISPR/AapCas12b system catalyzes cleavage of the ssDNA probe, leading to the dissociation of FAM-tagged gold nanoparticles from the probe’s biotinylated region.

Thus, FAM-gold nanoparticles pass through the streptavidin-coated C line, migrate forward, and are captured by T line-immobilized secondary antibodies, yielding a positive gold band (Figure 3A). Conversely, in negative samples, the intact ssDNA reporter probe is not subject to cleavage and is therefore completely retained by streptavidin-functionalized C line (Figure 3A). Our results demonstrated that the LFS T line gold signal was detected only when AapCas12b, crRNA, *B646L* gene, and gold particle probes were simultaneously present (Figure 3B). The detection outcomes of the AapCas12b-LFS assay showed complete concordance with those obtained via blue light, ultraviolet light, and fluorescence signal-based detection methods (Figure 3C,D).

### 3.5. Sensitivity of B646L-RPA-CRISPR/AapCas12b-LFS Method

**Figure 4:**
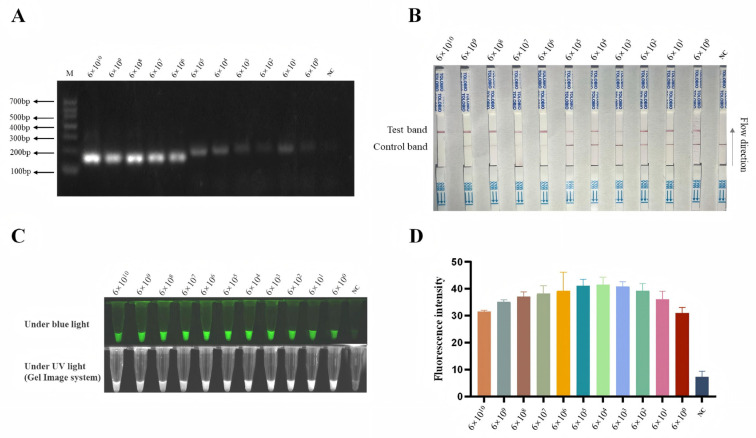
The sensitivity of *B646L*-RPA-CRISPR/AapCas12b-LFS detection. (**A**) RPA products of *B646L* amplified from pCE-*B646L* plasmid with different copy numbers. (**B**) The RPA products were detected by the CRSIPR/AapCas12b-LFS, and (**C**) The RPA products were detected by using blue light. (**D**) Green fluorescence signals were analyzed by ImageJ software.

To enhance the detection sensitivity of the CRISPR/AapCas12b system, we developed two RPA primers (Table 1) targeting the ASFV *B646L* gene before CRISPR/AapCas12b reaction (Figure 3A). Both pairs of RPA primers exhibited comparable performance in the *B646L* gene amplification, and the RPA primer 1 (RPA-*B646L*-F1/R1) was selected for subsequent experiments. Next, pCE-*B646L* plasmids were serially diluted 10-fold to obtain concentrations ranging from 6 × 10^10^ copies/µL to 6 × 10^0^ copies/µL. Each dilution was subjected to RPA, and the resulting products (Figure 4A) were subsequently detected using the AapCas12b-LFS system, with each dilution tested in triplicate.

The sensitivity of AapCas12b-based detection was assessed via LFS (Figure 4B) and cross-verified by using light and fluorescence detections (Figure 4C,D). As shown in the results, the RPA-AapCas12b-LFS system achieved a low limit of detection of 6 copies/µL (Figure 4B–D). The results suggested that the *B646L*-RPA-CRISPR/AapCas12b-LFS method has very high sensitivity for the detection of ASFV.

### 3.6. Specificity of RPA-CRISPR/AapCas12b-LFS Detection Method

**Figure 5:**
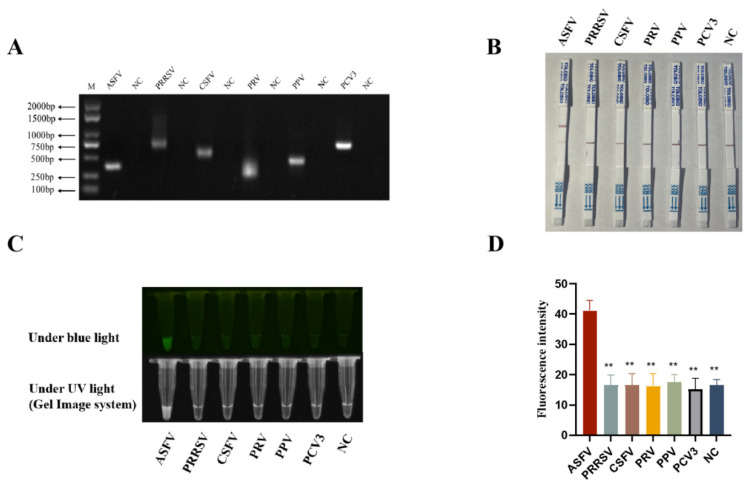
The specificity of RPA-CRISPR/AapCas12b-LFS detection. (**A**) The nucleic acid samples of five pig viruses and ASFV were all confirmed by RT-PCR for RNA viruses PRRSV and CSFV, and by PCR for DNA viruses ASFV, PRV, PPV4, and PCV3. (**B**) The RPA-CRISPR/AapCas12b detection specificity was examined by using LFS. (**C**) The RPA-CRISPR/AapCas12b detection specificity was verified by light detections. (**D**) Green fluorescence signals were analyzed by ImageJ software. ** *p* < 0.01 versus ASFV signal by Student’s *t* test.

In order to verify the specificity of RPA-CRISPR/AapCas12b detection method, nucleic acid samples of five pig viruses other than ASFV were obtained, including the RNA (RNA was converted into cDNA before detecting) from RNA viruses PRRSV and CSFV, and DNA from DNA viruses PRV, PPV4, and PCV3. The nucleic acid concentrations of ASFV, PPRSV, CSFV, PRV, PPV, and PCV3 samples are 57.2 ng/μL, 32.6 ng/μL, 183.6 ng/μL, 93.6 ng/μL, 76.3 ng/μL, and 75.3 ng/μL, respectively. The nucleic acid samples of five pig viruses and ASFV were all confirmed by PCR (Figure 5A) by using the specific PCR primers (Table 1). Results from the RPA-CRISPR/AapCas12b-LFS assay showed that gold particle signals were observed on the C lines of all porcine virus samples, whereas only the ASFV sample additionally presented positive gold particle signal on its T line (Figure 5B). Similar results were also obtained from RPA-CRISPR/AapCas12b reaction detection using blue and ultraviolet lights, and fluorescence detection (Figure 5C,D). Moreover, the specificity of the sgRNA/primers was verified via BLAST analysis (v.2.17.0), which confirmed exclusive targeting to ASFV genes. In summary, the RPA-CRISPR/AapCas12b method had very high specificity for detection of ASFV.

### 3.7. RPA-CRISPR/AapCas12b-LFS Detection of Clinical Samples

**Figure 6:**
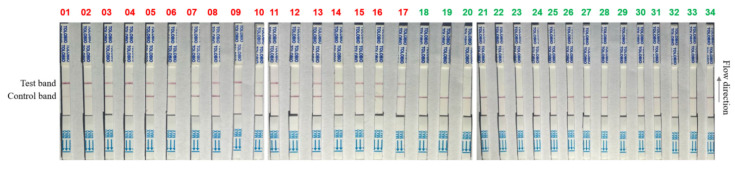
RPA-CRISPR/AapCas12b-LFS method for detecting a total of 34 clinical samples. A total of 17 samples (No. 1–17) were positive for ASFV, which are marked in red color, and 17 samples (Nos. 18–34) are negative for ASFV, marked in green color.

A total of 34 clinical samples were detected by using the established RPA-CRISPR/AapCas12b-LFS system. The results from RPA-CRISPR/AapCas12b-LFS showed that 17 clinical samples were positive (Nos. 1–17), and 17 clinical samples were negative (Nos. 18–34) (Figure 6). To validate the reliability of the CRISPR/AapCas12b-LPS assay in ASFV detection, verification was carried out by using qPCR, which is recommended by the WOAH (Table 1). The results of qPCR showed that the RPA-CRISPR/AapCas12b-LFS detection results were completely consistent with the qPCR detection results (Table 2), and the detailed information of qPCR Ct value is shown in (Supplement Table S1). These results further confirmed the reliability of the RPA-CRISPR/AapCas12b-LFS method for detecting clinical samples of ASFV.

## 4. Discussion

The CRISPR–Cas system has attracted growing attention as a diagnostic tool, driven by its specific gene targeting capability [15]. By combining Cas enzymes and gRNA for sequence-specific cleavage of target DNA/RNA, the CRISPR–Cas system has emerged as a powerful genetic engineering technique [16]. Beyond target specific binding and cleavage, Cas12a, Cas12b, and Cas13 exhibit trans-cleavage activity: upon Cas-gRNA-target complex formation, they non-specifically degrade surrounding single-stranded nucleic acids (ssDNA/ssRNA) [17,18]. Cas12a and Cas12b are both RNA-guided enzymes that target DNA, enabling their application in the detection of specific DNA sequences [19]. Cas13a is also an RNA-cleaving enzyme; however, its target is RNA, a characteristic that enables it to be used for the detection of specific RNA molecules [20,21]. Among these Cas protein systems, the most significant difference lies in their optimal reaction temperatures: Cas12a and Cas13b operate at 37 °C, whereas the Cas12b system functions at 60 °C [22]. Cas12b, derived from thermophilic bacteria, is highly thermostable and functions optimally at 60 °C and above [12]. The thermostability of Cas12b allows for high-temperature incubations, which can improve editing efficiency and reduce off-target effects [23].

Previously, our team has established two distinct RPA-CRISPR detection assays targeting the ASFV *D117L* and *S273R* genes, respectively, by coupling recombinase polymerase amplification (RPA) with CRISPR–Cas effector proteins: specifically, LbCas12a for *D117L* and LwCas13a for *S273R* [24,25,26]. Due to the different types of nucleic acids recognized by Cas12a and Cas13a, the combination of CRISPR/Cas12a and CRISPR/Cas13a systems may be used for simultaneous differential diagnosis of ASFV in a single tube. However, one study has suggested that the efficiency of Cas12a could be suppressed by Cas13a components when put together in a single reaction [27]. The reaction temperature of Cas12b is 60 °C, and it is possible that regulating different reaction temperatures to activate the activities of Cas12b and Cas13a, sequentially, and meanwhile, the high temperature of 60 °C can inactivate the enzymatic activity of Cas13a, thereby avoiding mutual interference. Thus, the aim of this study is to establish the RPA-CRISPR/AapCas12b method for detecting the ASFV. Also, it will lay the foundation for the future design of a method for simultaneous differential diagnosis of ASFV by combining the Cas12b and Cas13a detection systems.

Due to their critical biological functions and diagnostic utility, p72 (*B646L*), p30 (*CP204L*), p54 (*E183*L), and CD2V (*EP402R*) have been widely adopted as diagnostic targets for ASFV [6]. p72, as the major capsid protein, stands out for its highest conservation, making it the gold standard for genotyping via nucleic acid based methods like PCR, Loop-Mediated Isothermal Amplification (LAMP), and CRISPR–Cas system [6,28]. p30, an early–late structural protein, and p54, an envelope protein, are highly conserved and widely utilized in the detection of antigen and antibody [29,30]. The p30 protein is an early-expressed viral protein and excellent immunogenicity, which enables the detection of infected animals prior to the onset of clinical signs [29]. The p54 protein is widely recognized as a critical diagnostic target due to its unique biological properties and functional roles in viral infection [30]. CD2V exhibits genotype-specific variation and is predominantly associated with genotype II, thereby restricting its utility for detecting non-genotype II strains [31].

When comparing to other isothermal diagnostic techniques including RPA, LAMP, and Transcription-Mediated Amplification (TMA), CRISPR–Cas excels in specificity, multiplexing, and sensitivity, but is outperformed by RPA, LAMP, and TMA in speed and cost [32]. CRISPR–Cas systems have emerged as transformative tools in diagnostics, but they face critical limitations [33,34]. Firstly, a majority of CRISPR-based diagnostic platforms necessitate preliminary nucleic acid amplification, such as RPA or LAMP, to enhance the concentration of target sequences. However, these amplified amplicons exhibit high stability and may persist in laboratory settings, increasing the risk of cross-contamination and subsequent false-positive outcomes in downstream assays [35]. The core strategy for contamination prevention in CRISPR–Cas-based detection hinges on the integration of “physical isolation, strict spatial zoning, and full-process monitoring.” The incorporation of negative controls alongside environmental testing substantially mitigates the risk of false positives. For unexplained positive outcomes, priority should be given to investigating contamination sources in reagents, consumables, or operational workflows. Secondly, the pre-amplification step imposes practical constraints such as prolonged assay duration and reliance on complex instrumentation that compromise the accessibility and efficiency of CRISPR-based detection [36]. Thirdly, unlike qPCR, which offers broad applicability across diverse target sequences, the target recognition mechanism of Cas12 proteins is inherently dependent on PAM sequences. This PAM requirement restricts the range of detectable genetic regions in pathogen diagnostics, limiting the flexibility of CRISPR–Cas12 systems compared to qPCR [36].

In current research, a rapid, low-cost, and visual RPA-CRISPR/AapCas12b-LFS nucleic acid test for ASFV by selecting the conserved structural gene *B646L* was established. The method combines RPA and LFS and can be performed by non-specialists. The user only needs to add the required reagents to the nucleic acid sample to be tested, complete the reaction in a constant temperature water bath within 1 h, and observe the results with the naked eye. The reagents involved in the assay can be used for more than six months when stored between −15 °C and −25 °C. Water bath pots and pipettes are the main equipment required to perform testing, showing the potential application in on-site ASFV testing.

Meanwhile, we optimized the CRISPR/AapCas12b reaction parameters, including reaction time, probe, and sgRNA/AapCas12b ratio, and achieved optimal visualization not only under blue and UV light but also on lateral chromatography test strips. The detection sensitivity of RPA-CRISPR/AapCas12b-LFS can reach six copies. At the same time, the test showed no cross-reactivity with five other swine viruses, proving its excellent specificity, which is very important in clinical testing. In addition, each strip test costs about $0.61 according to our estimation. For the detection of 34 clinical samples, the coincidence rate of RPA-CRISPR/AapCas12b-LFS test with qPCR test was 100%, demonstrating its reliability in clinical diagnosis.

## 5. Conclusions

We have developed a rapid, sensitive, and specific RPA-CRISPR/Cas12b-LFS method for ASFV detection. It can be used for visual detection of ASFV in clinical samples and has potential significance for early monitoring, prevention, and control of ASF.

## Figures and Tables

**Table 1:** The information of primes.

Primer Names	Primer Sequences (5′-3′)
pET28a-AapCas12b-F	ATGGGTCGCGGATCCGAATTCATGGCCGTAAAATCTATGAAAGTTAA
pET28a-AapCas12b-R	GTGGTGGTGGTGGTGCTCGAGTCAGATGTCCCCAGTGTTCTCA
*B646L*-sgDNA-F	GAAATTAATACGACTCACTATAGGGGTCTAGAGGACAGAATTTTTCAACGGGTGTGCCAATGGCCACTTTCCAGGTGGCAAAGCCCGTTGAGCTTCTCAAATCTGAGAAGTGGCACCGTATCCGATCACATTACCT
*B646L*-sgDNA-R	AGGTAATGTGATCGGATACGGTGCCACTTCTCAGATTTGAGAAGCTCAACGGGCTTTGCCACCTGGAAAGTGGCCATTGGCACACCCGTTGAAAAATTCTGTCCTCTAGACCCCTATAGTGAGTCGTATTAATTTC
ssDNA-probe	5′-6-FAM-NNNNNNNNNNNN-BHQ1-3′
RPA-*B646L*-F1	CGCCATTATGCAGCCCACTCACCACGCAGA
RPA-*B646L*-R1	GATAAGATTGATACCATGAGCAGTTACGGA
RPA-*B646L*-F2	AGATTGGCACAAGTTCGGACATGTTGTTAACGCCA
RPA-*B646L*-R2	TAGTGGAAGGGTATGTAAGAGCTGCAGAACTTTGA
PRRSV-UF	GCCCCTGCCCAYCACG
PRRSV-UR	TCGCCCTAATTGAATAGGTGA
CSFV-UF	CTGGGTGGTCTAAGTCCTGAGTA
CSFV-UR	GATTCAACTCCATGTGCCATGTA
PRV-gC-F1	CGAGACCGAGGGCGTCTACAC
PRV-gC-R1	GCCCATCATCAGCGCCTGC
PPV4-F1	CTTTGCTTTGTCCAACGCAGA
PPV4-R1	TAGATGTCCTGGCACAGATACTTGAC
PCV3-F1	CTGTTATTTTGGATGATTTTTATG
PCV3-R1	CACAGCCGTTACTTCACCC
*B646L*/p72-F	CGGGTGCGATGATGATTACC
*B646L*/p72-R	TCTCTTGCTCTGGATACGTTAATATGAC
*B646*L/p72-TaqMan	5′-FAM-TCTCTTGCTCTGGATACGTTAATATGAC-BHQ1-3′
β-actin-F:	ATGAAGATCAAGGTGAGTGCC
β-actin-R:	TCGTACTCCTGCTTGCTGATC

Note: The T7 sequences are underlined.

**Table 2:** Comparison between RPA-CRISPR/AapCas12b-LFS and qPCR detections of clinical samples.

Sample Types	Samples Numbers	Detection Results (Positive Rates)	
		RPA-AapCas12b-LFS	qPCR
Heart	3	2/3	2/3
Liver	3	2/3	2/3
Spleen	3	2/3	2/3
Lung	3	2/3	2/3
Kidney	3	2/3	2/3
Lymph node	3	2/3	2/3
serum	5	1/5	1/5
Blood	5	2/5	2/5
Oral swab	6	2/6	2/6
Total	34	50%	50%

## Data Availability

Data are contained within the article and Supplementary Materials.

## Supplementary Materials

The following supporting information can be downloaded at: https://www.mdpi.com/article/10.3390/ani15223274/s1, Supplement Table S1: The information of Ct value, and ASFV strain.
