# Supplementary material for: Development of Visual Detection of African Swine Fever Virus Using CRISPR/AapCas12b Lateral Flow Strip Based on Viral Major Capsid Protein Gene B646L
Source: Animals (Basel). 2025 Nov 12;15(22):3274. doi: 10.3390/ani15223274 (PMC12649315; doi:10.3390/ani15223274)
Supplement: Supplementary file 1 [file animals-15-03274-s001.zip › animals-3921185-supplementary.pdf]

Supplement Table S1: The information of Ct value, and ASFV strain

Sample	Sample type	B646L Ct value	ASFV Genotype	Swine β-actin Ct value
1	Heart	19.85	Genotype II	23.22
2	Heart	23.1	Genotype II	26.27
3	Liver	19.97	Genotype II	29.05
4	Liver	19.25	Genotype II	29.38
4	Spleen	20.42	Genotype II	24.53
6	Spleen	22.32	Genotype II	30.73
7	Lung	34.83	Genotype II	27.45
8	Lung	19.41	Genotype II	30.31
9	Kidney	32.73	Genotype II	24.46
10	Kidney	34.26	Genotype II	23.82
11	Lymph node	22.52	Genotype II	24.87
12	Lymph node	33.71	Genotype II	25.64
13	serum	36.28	Genotype II	27.63
14	Blood	31.23	Genotype II	23.2
15	Blood	29.92	Genotype II	23.24
16	Oral swab	28.98	Genotype II	26.92
17	Oral swab	26.57	Genotype II	22.92
18	Heart	N/A		29.27
19	Liver	N/A		29.63
20	Spleen	N/A		23.62
21	Kidney	N/A		25.32
22	Lymph node	N/A		26.72
23	serum	N/A		30.95
24	serum	N/A		35.21
25	serum	N/A		32.9
26	serum	N/A		33.72
27	Blood	N/A		22.76

28	Blood	N/A		26.77
29	Blood	N/A		23.82
30	Blood	N/A		25.85
31	Oral swab	N/A		27.46
32	Oral swab	N/A		28.37
33	Oral swab	N/A		29.41
34	Oral swab	N/A		28.81
35	Extraction control	N/A		N/A
36	Amplification control	N/A		N/A
